# A Hemoglobin Variant Causing an Unexplained Low Oxygen Saturation by Pulse Oximetry: Two Case Reports

**DOI:** 10.7759/cureus.42182

**Published:** 2023-07-20

**Authors:** Inês B Rua, Beatriz Vala, Inês Gameiro, João R Martins, Rui Castelo

**Affiliations:** 1 Neonatology Department, Maternidade Daniel de Matos, Centro Hospitalar e Universitário de Coimbra, Coimbra, PRT; 2 Pediatrics Department, Centro Hospitalar de Leiria, Leiria, PRT

**Keywords:** neonatology, hematology, hemoglobinopathies, pulse oximetry, oxygen saturation

## Abstract

Pulse oximetry is now routinely used in neonatal resuscitation and for neonatal screening for congenital heart diseases. Beyond respiratory and cardiac diseases, hemoglobin (Hb) variants must be included in the differential diagnosis of low oxygen saturation detected by pulse oximetry. We aim to describe two cases of fetal Hb variant (heterozygous γ-globin gene (HBG1) mutation in exon 2 c.202G>A (p.Val68Met)), which was identified in two unrelated newborns.

## Introduction

Pulse oximetry is a non-invasive spectrophotometric method used to estimate arterial oxygen saturation. The oximeter device consists of a light emitter and a detector placed on a person’s skin; it uses two light wavelengths - red (660nm) and infrared (940nm) - and it relies on the principle that oxyhemoglobin and reduced hemoglobin (Hb) have different light absorption properties [1]. Deoxyhemoglobin is characterized by greater red-light absorption, whereas oxyhemoglobin has higher absorption in the infrared spectrum [1]. The ratio of absorbance at these wavelengths is then calculated and adjusted to direct measurements of arterial oxygen saturation (SaO2) to establish the oxygen saturation by pulse oximetry (SpO2) [2]. This instrument plays an important role in pediatrics and neonatology, as it can detect changes in oxygen delivery and consumption and is commonly employed in neonatal resuscitation and neonatal screening for congenital heart diseases [1].

However, pulse oximetry has limitations, including probe positioning, motion artifacts, time lag, low perfusion states, irregular rhythms, in anemic people, and the presence of abnormal Hb molecules [1,2].

Low SpO2 may be found not only in diseases of the respiratory tract (e.g. primary ciliary dyskinesia), functional lung diseases (e.g. hyaline membrane disease), and congenital heart diseases (e.g. Tetralogy of Fallot) but also in the presence of Hb variants, in which PaO2 and SaO2 in arterial blood gas (ABG) samples are normal, but SpO2 is low [3-5].

Hb, a tetrameric protein, contains two α-globin chains and two non-α-globin chains (β, γ, δ) that combine with four heme groups. In adults, normal Hb predominantly consists of HbA (α2β2), with a small portion of HbA2 (α2δ2) and an even smaller portion of fetal Hb (HbF, α2γ2). In newborns, HbF accounts for the largest fraction, and the transition to HbA occurs within the first six months of age [5].

Hemoglobinopathies occur when genetic alterations lead to Hb variants, due to the synthesis of a Hb with an abnormal structure, or in thalassemias, due to reduced synthesis of globin chains [3,6,7].

There are 1,864 hemoglobinopathies, 1,424 of which consist of Hb variants [8]. Mutations in HBG1 or HBG2, γ-globin genes, can cause fetal and neonatal symptoms that are temporary and will disappear in the first months of life. Mutations in HBA1 or HBA2, α-globin genes, can cause newborn cyanosis that will persist throughout life. Mutations in HBB, the β-globin gene, will only become clinically significant a few months after birth, as the switch from γ-globin to β-globin ensues [9].

To establish a diagnosis of hemoglobinopathy, we may need an ABG sampling to document PaO2 and rule out methe/carboxyhemoglobinemia. If PaO2 is normal, further evaluation of Hb is required, such as Hb electrophoresis and high-performance liquid chromatography. If an abnormal Hb variant is identified, DNA sequence analysis is necessary to detect and characterize the specific mutation [5].

We describe two cases of a Hb variant, caused by mutations in HBG1 exon 2 c.202G>A (p.Val68Met), a mutation that has been reported only once to date [3].

## Case presentation

Case number one

**Figure 1:**
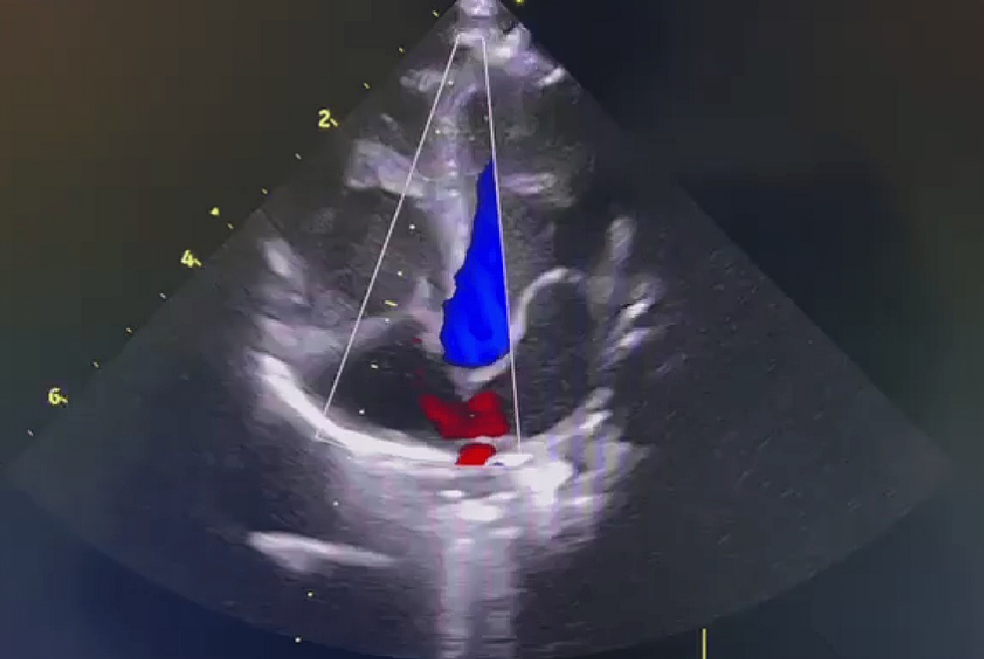
Echocardiogram of case number one Echocardiogram of case number one showing a small ostium secundum-type interatrial communication with an exclusive left-to-right shunt

A male-term newborn with no relevant family history was delivered by cesarean due to fetal distress. He had an Apgar score of 9 at one minute and 10 at five minutes, weighed 2910g, and the physical examination was unremarkable. In the first hour of life, he developed tachypnoea, nasal flaring, and low SpO2 (88%), so he was transferred to the neonatal intensive care unit (NICU) and started supplemental O2 and nasal CPAP due to the clinical suspicion of transient tachypnea of the newborn. Signs of respiratory distress resolved, but his SpO2 remained low. There was no gradient between pre- and post-ductal SpO2 and blood pressure, and his chest X-ray was normal. An echocardiogram revealed a small ostium secundum-type interatrial communication with an exclusive left-to-right shunt (Figure 1). He did not show any signs of respiratory distress during hospitalization, but had persistent low SpO2 (minimum of 80% during wakefulness), with no response to supplemental oxygen. Antibiotic therapy was administered for three days, and septic screenings were negative. ABG analysis was performed at room air: pH of 7.41, pO2 of 80mmHg, pCO2 of 38mmHg, HCO3 of 24mmol/L, and normal lactate. The Hb study was normal, identifying 84% of HbF. Meanwhile, a molecular study identified heterozygosity for c.202G>A(p.Val68Met) variant, located in exon 2 of the HBG1 gene, already described in association with transient neonatal cyanosis. During hospitalization, he repeated an echocardiogram that was normal. He was discharged on day 23 of life and referred to a hematology consultation. At one month of age, he repeated high-performance liquid chromatography that showed 32% of HbF, and pulse oximetry revealed normal SpO2 values.

Case number two

**Figure 2:**
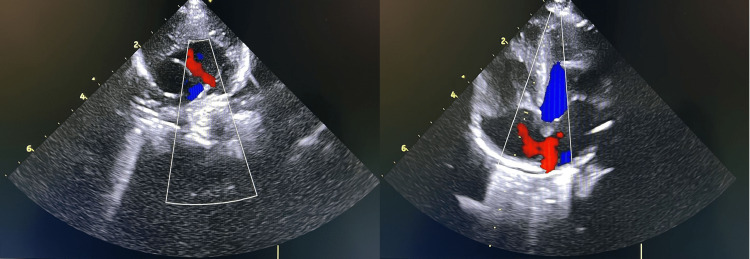
Echocardiogram of case number two Echocardiogram of case number two showing a patent foramen ovale (right) and a small ductus arteriosus (left), both with a left-to-right shunt

A Caucasian female-term neonate was born by assisted (ventouse) vaginal delivery due to fetal distress. She had an Apgar score of 9 at one minute and 10 at five minutes, weighed 2260g, and the physical examination was normal. Parents were consanguineous. Congenital cardiopathies screening at 58 hours of life revealed no gradient between pre- and post-ductal SpO2, but values ranged between 88% and 89%, with no response to the hyperoxia test, so she was admitted to the NICU. The echocardiogram showed a patent foramen ovale and a small ductus arteriosus, both with left-to-right shunt (Figure 2). The septic screening was negative, and the ABG at room air showed pH of 7.41, pO2 of 97.1mmHg, SaO2 of 97%, pCO2 of 44.1mmHg, HCO3 of 29mmol/L, and lactate of 1.7mmol/L. The Hb study was normal, with 82.7% of HbF. A molecular study identified a mutation on exon 2 of the HBG1 gene, revealing heterozygosity for the c.202G>A (p.Val68Met) variant. She was discharged on day six of life without any complications during the hospital stay. An appointment was scheduled by day 12, maintaining the SpO2 values of 86-87%. At three months of age, she repeated high-performance liquid chromatography that showed 15% of HbF, and pulse oximetry revealed normal SpO2 values.

## Discussion

The use of pulse oximetry in neonatal resuscitation and neonatal screening for congenital heart diseases enables the detection of various medical conditions, including not only respiratory and cardiac diseases but also hemoglobinopathies. When confronted with unexplained low peripheral capillary oxygen saturation (SpO2), particularly in clinically asymptomatic individuals, it is crucial to consider hemoglobinopathies as part of the differential diagnosis. This consideration helps avoid unnecessary investigations since certain Hb variants can lead to inaccurate readings when using pulse oximetry. A normal measurement of SaO2 on arterial blood gas samples, measured by CO-oximetry, will demonstrate the lack of hypoxemia and suggests the need for testing for a Hb variant [4].

With these two cases, we aim to raise awareness among clinicians about the existence of this particular Hb variant, only described once previously [3], that may cause low SpO2 but normal SaO2 and PaO2. In newborns, HbF constitutes the largest fraction of Hb [5], and this mutation only affects γ-globin and, therefore, only affects fetal Hb [9]. As HbF decreases over time, usually in the first four to six months of age, and the transition to HbA occurs, a Hb with two α chains and two δ chains (no γ chains), and saturation values improve [9].

Moreover, this Hb variant seems to be associated with high oxygen affinity, which may compromise oxygen delivery to tissues and cause mildly elevated lactate [3], something not present in our cases.

Interatrial communication is present in most neonates, mostly due to patent foramen ovale and in a minority due to a true atrial septal defect of “the ostium secundum” type [10]. As case number one had an exclusive left-to-right shunt, it could not be responsible for low oxygen saturation. Although they were present in our causes, an association between this mutation and interatrial communications was not previously described.

We also want to emphasize the importance of molecular study in establishing the diagnosis of a Hb variant and allowing accurate genetic counseling, ultimately preventing unnecessary exams and, in this particular mutation, predicting an excellent long-term prognosis (resolution at four to six months).

## Conclusions

Neonatal screening for congenital heart diseases plays an important role in the early recognition of potentially life-threatening disorders. Although it is a rare disease, Hb variants must be included in the differential diagnosis of an unexplained low SpO2 measurement, when there is a discrepancy between the ABG and the pulse oximeter, especially in otherwise well newborns.
